# Preparation and Characterization of Rivastigmine Transdermal Patch Based on Chitosan Microparticles

**Published:** 2016

**Authors:** Mohsen Sadeghi, Fariba Ganji, Seyyed Mojtaba Taghizadeh, Bahram Daraei

**Affiliations:** a *Biomedical Engineering Group, Faculty of Chemical Engineering, Tarbiat Modares University, Tehran, Iran.*; b *Novel Drug Delivery Systems Department, Polymer Science Faculty, Iran Polymer and Petrochemical Institut, Tehran, Iran. *; c *Department of Toxicology, Faculty of Medical Science, Tarbiat Modares University, Tehran, Iran.*

**Keywords:** Spray drying, Transdermal patch, Rivastigmine, Drug-in-adhesive, Alzheimer’s disease, Chitosan microparticles

## Abstract

Here we report a novel approach for preparation of a 6-day transdermal drug delivery system (TDDS) as treatment for mild to moderate Alzheimer’s disease. The spray drying method was used to prepare microparticles containing the anti-Alzheimer drug, Rivastigmine, in combination with the natural polymer, chitosan, for transdermal drug delivery applications. The content of the drug was determined by High Performance Liquid Chromatography (HPLC) method which was validated as per FDA guidelines. The morphology and size range of the microparticles were determined; and the effect of drug concentration in the solution injected into the spray dryer on the particles characterizations was studied. The stability of Rivastigmine at high temperature was confirmed using FTIR analysis as well as a validate HPLC assay. The obtained results show that the drug was stable at high temperatures with 7 to 42% loading in the microparticles, and the higher drug concentrations of the solution injected into the spray dryer resulted in increase of the drug loading, surface drug and microparticles distortion. The TDDS containing the microparticles was also prepared with microparticle to dry adhesive ratios of 5, 10 and 15% using acrylic adhesive. Based on adhesion properties of the patches, gained from the probe tack and the peel adhesion 180° tests, and the 15% patch by having more drug content per unit area of the patch, and still having similar adhesion properties was compared to the microparticles-free patch of 5.1% Rivastigmine salt (equivalent to the drug content of the 15% patch) from the permeation point of view by using Franz cell diffusion over 6 days. The drug permeation rate from the microparticle-free patch was slower than the 15% microparticles patch, which is the result of crystallization of Rivastigmine salt in the acrylic adhesive. The 6-day-prepared TDDS can be considered as an alternative for one-week application of 6 Exelon patches.

## Introduction

Alzheimer›s disease (AD) is a progressive brain disorder which decreases the patient›s mental ability and causes loss of mental, behavioral, functional, and learning abilities. More than 80% of AD cases worldwide are aged people (1, 2).

Among aged populations (older than 65 years), the percentage of AD is doubling every five years; about 200,000 people younger than 65 years with AD comprise the younger onset AD population and 5 million are 65 years old or more. It is predicted that by 2050, the overall estimated prevalence is expected to be 13.8 million and one new case will join the AD population, every 33 seconds (3). 

Various hypotheses have been proposed to explain the AD mechanism. The oldest hypothesis, which is the foundation of the most recent existing drug therapies, is known as the cholinergic hypothesis. According to this hypothesis, the AD is the result of a deficiency in the synthesis of acetylcholine in the human body (4).

The conventional treatments for the management of AD have all been administered orally. Some of the adverse effects of cholinesterase inhibitors, such as donepezil, galantamine, physostigmine, tacrine, phencerine and Rivastigmine that occur and often lead to termination of treatment by patients, are hepatotoxicity, renal failure and asthenia or malaise (4, 5). Transdermal therapy has the potential to decrease the side effects of oral administration of drugs and providing easier access to optimal doses, benefits which would improve patient compliance (6, 7).

So far, transdermal drug delivery systems (TDDSs) containing acetylcholinesterase inhibitor agent have been suggested. For example, development of transdermal Tacrine have been used for a while in the clinical stage, but is no longer used clinically; the reason was that local irritation occurred in all patients and it had hepatotoxicity potential when used for the long-term management of AD. physostigmine TDDS was proposed and evaluated. The high degree of inter-subject variation and the observation of no reliable relationship between different dosages and efficacy show that the patch did not sufficiently compensate for the lack of acetylcholine in the areas of the brain that control cognition, behavior and emotion. Transdermal formulation of phenserine tartrate has also been developed, characterized and arrived to clinical stages which is still ongoing. Among these, only the Rivastigmine transdermal patch has been commercialized in the form of a daily matrix type patch which is known in the market as Exelon^®^ Patch (4).

Rivastigmine is a reversible cholinesterase (acetylcholinesterase and butyrylcholinesterase) inhibitor for the treatment of mild to moderate alzheimer disease. Rivastigmine hydrogen tartrate (RHT) with a molecular weight of 400.43 g, is a white to off-white, fine crystalline powder that is very soluble in water, soluble in ethanol, very slightly soluble in ethyl acetate (8, 9).

Chitosan, an N-deacetylated derivative of chitin, is a most frequently natural polymer in biomedical area having structural possibility for chemical and mechanical modifications to generate novel properties, functions and applications (10). In recent years, chitosan has been used for development of drug delivery systems; for example, it has been used for the preparation of drug carriers and reservoirs in controlled release systems (11).

The objectives of the present study are to prepare a Rivastigmine TDDS, which unlike existing commercial product, is a drug-in-adhesive TDDS and lasts more than one day. The preparation process of the drug-in-adhesive is easier than matrix systems (e.g. Exelon^®^ patch). For this purpose, Rivastigmine loaded chitosan microparticles were prepared using the spray drying method. The physicochemical properties of the microspheres related to preparation parameters, and release characteristics *in-vitro* were studied.

Transdermal drug delivery system containing the microparticles was prepared. Adhesion properties, *in-vitro* permeation and morphological properties of microparticles in the adhesive were also evaluated.

## Experimental

*Materials*

Medium molecular weight chitosan was purchased from sigma-Aldrich (USA). High performance liquid chromatography (HPLC) grade acetic acid, ethanol, ethyl acetate, acetonitrile and analytical grade di ammonium hydrogen phosphate, were purchased from Merck (Germany). Rivastigmine hydrogen tartrate (RTH, Mw=400.43 g/mol) was received as a gift sample from Towfigh Pharmaceutical Ltd, (Tehran). Acrylic adhesive (Duro-Tak 2582), polyethylene CoTran 9720 as a backing layer and the SC45 release linear were procured from Henkel Lte, Germany, 3M Lte, USA and Bordarshib Lte, IRAN, respectively.

*Microparticles preparation*

The required volume of 1% solution of low molecular weight chitosan in 1% (v/v) acetic acid was prepared and stirred overnight. Different amounts of RHT was added to the chitosan solution to reach the RHT to chitosan mass ratios of 10% (group 1), 50% (group 2) and 100% (group 3). The resulting solutions were passed through a 22 µM Control Biogen filter, then injected to the spray drier (Büchi-190, Germany). Spray drying was done using a 1mm nuzzle. The processing conditions were as follows: inlet and outlet temperatures 150 and 75 °C, feed rate 3 mL/min, aspiration rate 75% and air flow 700 l h. These conditions were selected based on preliminary experimentation. The powders were removed from the collection vessel, sealed in 35 mm petri dishes and placed in desiccators over silica gel at room temperature until analysis (12, 13).

*Determination of drug content*

The amounts of RHT in the three groups of microparticles were determined using the HPLC method. Samples of dry powder formulations (1 mg) were dissolved in 10 mL of 1% (v/v) acetic acid. The drug contents in the microparticles were calculated using the fallowing equation (12). All the measurements were triplicated and averaged:

 Loading Capacity LC=Drug Content Microparticles Weight×100

(1)

Since the amount of drug at the surface of microparticles (surface drug) plays an important role in the drug burst release, the free drug contents were evaluated. For this purpose, considering high water swelling ratio of chitosan microparticles, ethanol was used due to its high solubility of RHT and insolubility of chitosan microparticles. Certain amounts of the three groups of microparticles were dispersed in 10 mL pure ethanol, then centrifuged at 1000 rpm and 25 °C for 5 min, and the supernatants were analyzed using HPLC to determined surface drug contents. The surface drug contents of microparticles were calculated as per equation given below with all the measurements performed in triplicate and averaged (12):

(2) Surface Drug=Ethanol Drug Content Total Drug Content×100

*Characterization of chitosan microparticles*

The chitosan microparticles morphology was evaluated by a S1460 field emission scanning electron microscope (Hitachi, Germany). Range of microparticles size was measured by Image J analysis for FESEM of microparticles. Potential degradation of RHT due to using high temperature in the spray drying process was evaluated by FTIR analysis.

*In-vitro drug release*

The *in-vitro* release profile of RHT from three groups of microparticles were performed in 2 mL vials containing 1 mL pure ethanol as release medium. The RHT was highly soluble in ethanol, while chitosan microparticles were insoluble in ethanol, that’s why the ethanol was used as release medium. Certain amounts of each group of microparticles were added to vials. At selected time intervals, release medium was removed completely and replaced by the same amount of fresh ethanol. This procedure was performed three times for each group. The samples were analyzed using HPLC.

*TDDS preparation*

The RHT TDDS was consisted of a backing layer (Polyethylene monolayer film), on which were applied successively a drug-in-adhesive layer covered by a protective release liner.

*Preparation of drug-in-adhesives mixtures*

The drug-in-adhesive mixtures were prepared in two groups, one of which containing drug loaded microparticles (MPL group) and the other had RHT, but no microparticles (RHT group). In order to prepare the MPL group with microparticle to dry adhesive ratios of 5, 10 and 15%, certain amounts of microparticles were dispersed in ethyl acetate using ultrasonic bath. Then acrylic adhesive was added and the resulting mixture was ultrasonicated and stirred overnight.

The second mixture contained the RHT salt, instead of MPL. In this case the salt was dissolved in ethanol using ultrasonic bath. Then acrylic adhesive was added and the same procedure repeated.

*Transdermal patches preparation*

Preparation of Rivastigmine patches was carried out in a way that the final patches have a thickness around 80 µM. For this purpose, the RHT and MPL group mixtures were spread by film applicator (Elcometer 3580, America) at laboratory temperature on the polyethylene backing layer. The drug-in-adhesives films (MPL and RHT patches) were dried at room temperature for 30 min, and subsequently at 60 °C for 1 h. Then the release liner was applied to the surface of the adhesive layer using a standard roller.

*Morphological evaluation of microparticles in the adhesive*

The morphology of microparticles in adhesive was evaluated by FESEM. For this purpose, group 2 of microparticles was dispersed in acrylic adhesive (in a way that forms microparticle to dry adhesive ratio of 50%) and spread as previously described. Morphological studies of microparticles in adhesive were carried out using FESEM imaging of cross section and surface of the film.

*Adhesion properties*

Adhesion properties of the prepared transdermal patches (MPL and RHT patches) were studied using peel adhesion 180° and probe tack tests. According to results of MPL patches adhesion properties, one of them was selected for further evaluations.

*Peel adhesion 180° test*

One week after the preparation of TDDSs, they were cut into 2.5 cm wide and 25 cm long strips. The tests were carried out using an Adhesion/Release Tester (Ar-1000, Chem Instruments Fair- Field, America) according to ASTM D3330. The strips were applied to a clean stainless steel plate, smoothed with a standard roller five times and pulled from the plate at a rate of 30 cm min^–1 ^after 20 minutes of rest time. The forces were expressed in N dm^-1^ width of adhesive tape. Each test was performed in triplicate and averaged (14-16).

*Probe tack test*

Tack is defined as the ability to instantaneously stick to a substrate under low pressure and be easily removed by adhesive separation (without leaving any residue at the substrate surface). One week after preparation of TDDSs, they were cut into 2 cm × 2 cm pieces. The tests were performed by a Probe Tack machine (PT-500, Chem Instruments Fair- Field, America) according to ASTM D2979. The probe was pressed lightly on the patch at the rate of 10 mm s^-1^ for about 1 sec and then withdrawn with the same rate. The adhesion was measured based on maximum force and expressed in N mm^-2^. The tests were replicated six times and averaged (14-16).

*In-vitro permeation studies*

Selected MPL patch and the RHT patch were evaluated by *in-vitro* permeation study. In vitro permeation of the patches were determined using a 5 mL Franz diffusion cell apparatus. The tests were done on Cellulose filter membrane (Sartorius, Germany) of 0.22 µM pore size as a diffusion membrane. After a week of rest for the TDDSs, they were cut into pieces with a 13 mm diameter and placed on the surface of the membrane that was clamped in between the donor and the receptor chamber of the diffusion cell. The receptor medium (7.4 phosphate buffer) was stirred by magnetic bar at temperature 37 ± 1 °C. At predetermined time intervals, 5 mL sample were withdrawn from the receptor compartment and were replaced immediately by equal volume of the fresh buffer solution. The collected samples were then passed through a syringe filter (pore size 0.45 µM, Millipore, USA). The drug content of the filtered solution was estimated using HPLC at 217 nm (70). The tests were repeated three times for each sample.

*High performance liquid chromatography*

*Chromatographic conditions*

Optimal conditions were obtained based on previous studies and pre-experiments in order to gain the best curve fit. The Agilent-1200 HPLC device equipped with a reversed-phase C18 analytical column (25cm × 4.6 i.d., 1.8 μM) was performed at 50 °C. The mobile phase, consisting 1mM di ammonium hydrogen phosphate buffer (pH = 2) and acetonitrile (75/25 v/v), was delivered at a rate of 1mL/min. the the peak area versus concentration data was treated by least-squares linear regression analysis. Wavelength of UV detection was 217 nm (17).

*Evaluation of analytical method*

Estimation of Rivastigmine in dosage forms by HPLC method was carried out using optimized chromatographic conditions. The method was validated as per FDA guidelines.

*Linearity*

Linearity test solutions for the assay method were prepared from Rivastigmine hydrogen tartrate stock solution at three concentration ranges (0.15-5, 5-300 and 300-1000 µg/mL) (18-20). 

*Precision*

The precision of the method was demonstrated by inter day and intra day variation studies. The various standard solutions were prepared in each concentration range. The intra day studies were performed by three repeated injections of each standard solution. 

The percentage relative standard deviation (%R.S.D) and the percentage recoveries were calculated. In the inter day variation studies, three repeated injections of each standard solution were made for three consecutive days and the percentage R.S.Ds and recoveries were calculated (18-20).

*Accuracy*

The accuracy of the assay method was evaluated in triplicate at various concentration levels for each range. The % recoveries and %R.S.D were calculated from fallowing equations:

$\mathrm{\% R.S.D}=\frac{Ca-Cm}{Ca}\times 100$

(3)

$\mathrm{\% Recovery}=\frac{Cm}{Ca}\times 100$

(4)

Were C_a_ and C_m_ are actual and measured concentrations, respectively (18-20).

*Limit of detection (LOD) and limit of quantification (LOQ)*

The LOD and LOQ of the developed method were determined by injecting progressively low concentrations of the standard solutions. LOD and LOQ were calculated using the following equations:

$\mathrm{LOD}=\frac{3\mathrm{SD}}{M}$

(5)

$\mathrm{LOQ}=\frac{10\mathrm{SD}}{M}$

(6)

Where SD is standard deviation of the response and M, the slope of the calibration curve (18-20).

*Statistical analysis*

The tests were repeated at least three times for each samples. All data are reported as mean ± SD.

**Figure 1:**
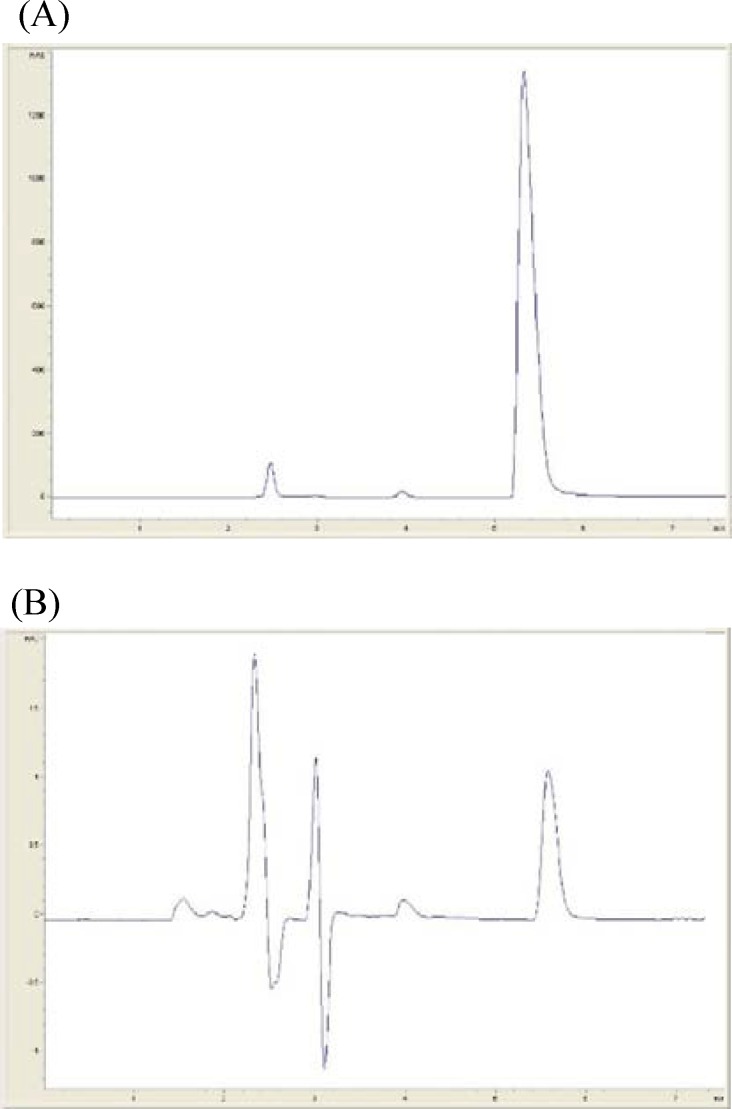
Typical HPLC chromatograms of Rivastigmine hydrogen tartrate: (A) 1500 µg/mL and (B) 1 µg/mL.

**Figure 2.**
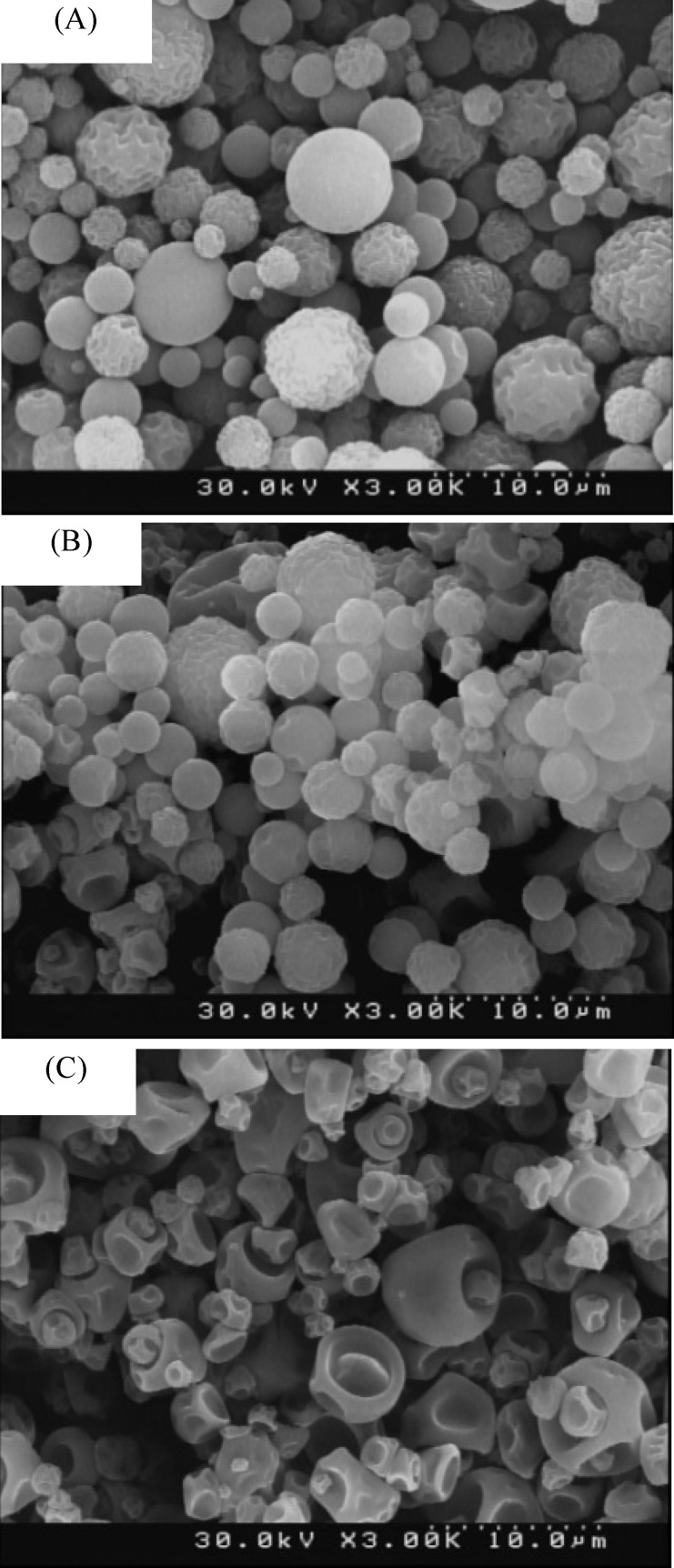
FESEM images of the microparticles for (A) group 1, (B) group 2 and (C) group 3.

**Fig. 3:**
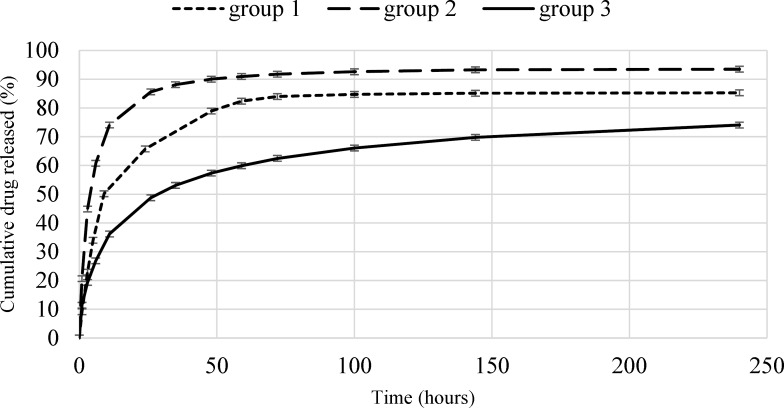
Cumulative release profiles of Rivastigmine from all groups of microparticles

**Fig. 4:**
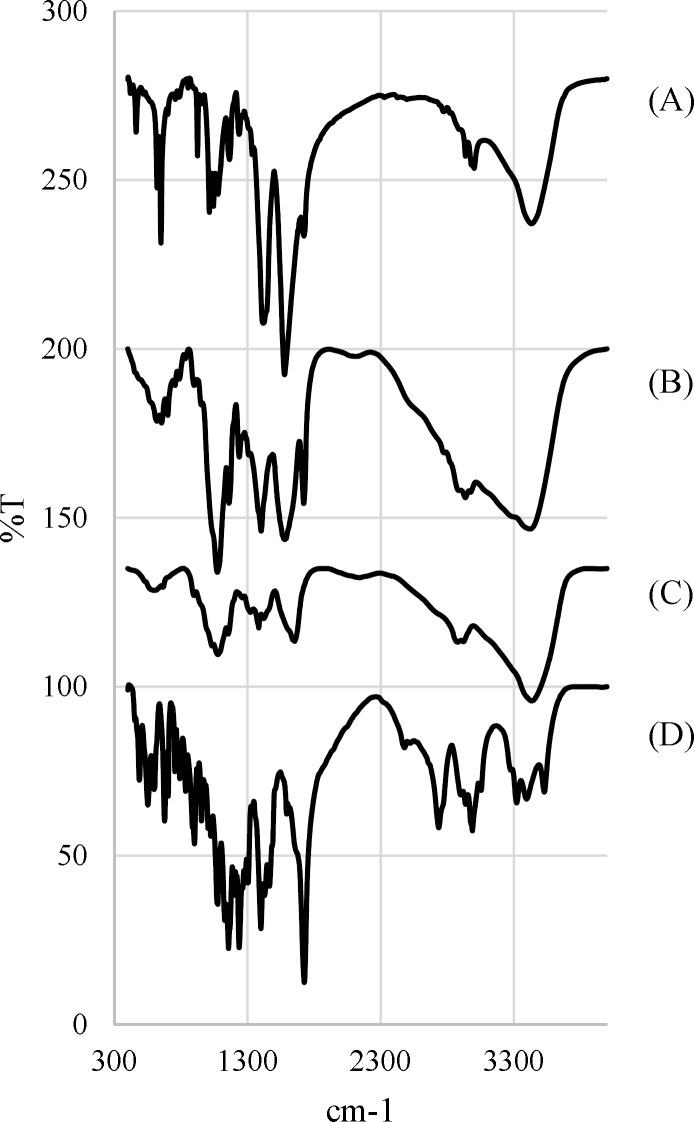
FTIR spectrums of (A) freeze-dried chitosa solution n-drug, (B) microparticles, (C) Chitosan and (D) Rivastigmine Hydrogen Tartrate

**Figure 5:**
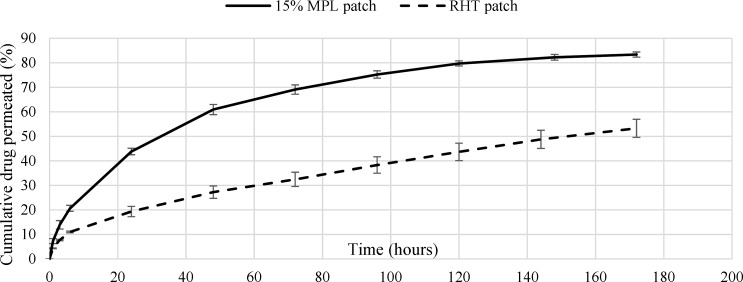
Cumulative permeation profiles of Rivastigmine from 15% MPL and RHT patches

**Fig. 6:**
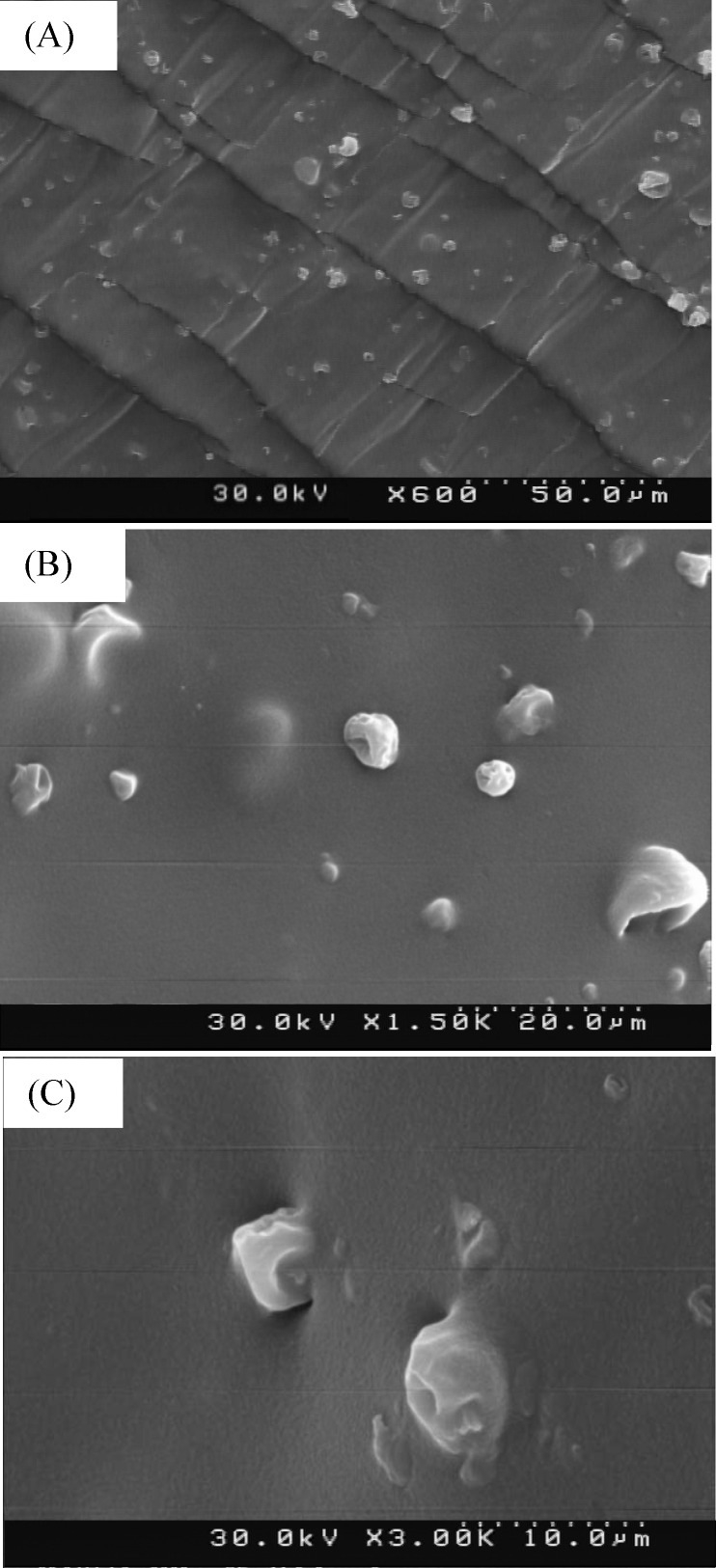
FESEM images of dispersed group 2 of microparticles in the acrylic adhesive for (A) cross section (600x), (B) surface (1500x) and (C) magnified surface (3000x

**Table 1:** Accuracy results of Rivastigmine

**Actual Concentration** **(µg/mL)**	**Measured**		
	**Mean±SD** **(n=3)**	**Recovery (%)**	**R.S.D** **(%)**
0.2	0.17±0.00	87.59	12.40
0.4	0.35±0.00	88.58	11.41
0.8	0.73±0.02	91.51	8.48
8	7.19±0.08	89.90	10.09
40	38.52±0.46	96.30	3.69
80	79.75±0.71	99.68	0.31
200	207.25±7.89	103.62	3.62

**Table 2:** Inter day precision results of Rivastigmine.

**Actual Concentration ** (μg/mL)	**Measured**		
	**Mean±SD (n=3)**	**Recovery (%)**	**(%) R.S.D**
0.15	0.13±0.00	91.55	8.44
0.5	0.46±0.02	93.73	6.26
1	0.97±0.06	97.28	2.71
5	4.85±0.22	97.11	2.88
20	18.90±0.33	94.53	5.46
100	99.32±2.93	99.32	0.67
500	506.13±7.05	101.22	1.22

**Table 3:** Intra day precision results of Rivastigmine

**Actual Concentration (µg/mL)**	**Measured**		
	**Mean±SD (n=3)**	**Recovery (%)**	**(%) R.S.D**
0.15	0.16±0.00	111.88	11.80
0.25	0.26±0.00	105.58	4.96
0.5	0.52±0.01	104.96	5.58
1	1.00±0.01	100.22	0.22
2	2.00±0.03	100.28	0.28
3	3.02±0.00	100.84	0.84
5	5.02±0.04	100.51	0.51
10	10.11±0.04	101.10	1.10
20	18.65±0.03	93.28	6.70
60	59.31±0.59	98.86	1.10
100	100.31±0.53	100.31	0.31
300	296.95±1.14	98.98	1.01
500	505.54±3.42	100.10	1.10

**Table 4:** The results of drug loading and surface drug of the microparticles

groups	Drug concentration of the initial solution (mg/mL)	Drug loading (%)	Surface drug (%)
**1**	1	7	1.7
**2**	5	9	2.7
**3**	10	42	7.8

**Table 5:** The results of the evaluation morphology and he microparticles size

groups	The concentration of the initial solution (mg/mL)	Drug to polymer ratio of the initial solution (%)	Folds of microparticls	microparticls size range (µm)
**1**	1	10		1-5
**2**	5	50	**	1-5
**3**	10	100	***	1-5

* Indicates the amount of distortion on the surface of the microparticles.

**Table 6:** Adhesion properties of MPL and RHT patches

TDDSs		Probe tack test (N/mm^2^)	Peel adhesion 180° test (N/10mm)
**MPL patches**	15% patch	3.47±0.92	12.19±0.36
	10% patch	3.70±0.77	12.41±0.57
	5% patch	3.81±0.48	13.03±0.79
**RHT patch**		5.04±0.26	16.60±0.38

transdermal drug delivery System.

## Results and discussion

*High performance liquid chromatography*

Typical chromatograms of sample solution of rivastigmine in high and low concentration (1500 and 0.1 µg⁄mL repectively) are shown in Figure 1. The retention time of Rivastigmine was found to be 5.5 min.

*Linearity*

The calibration curves for assay method were obtained over the concentration ranges. According to 7, 8 and 9 linear regression equations, the correlation coefficients were higher than 0.999. The results show that an excellent correlation exists between the peak area and drug concentration.

**Rivastigmine**

concentration (0.15-5μ g/mL) = 0.1003× peak area - 0.0194

R^2 ^= 0.9999                     (7)

**Rivastigmine**

concentration (5-300 µg/ml) = 00.1001× peak area - 1.1154

R^2 ^= 0.9998                     (8)

**Rivastigmine**

concentration (300-1000 µg/ml) = 0.1043× peak area - 1025

R^2 ^= 1                     (9)

*Accuracy*

The accuracy of the method was determined by %recovery and %R.S.D standards (Table 1.). The maximum %R.S.D of accuracy studies was 12.40%. The percentage recovery of Rivastigmine hydrogen tartrate was between 87.59 and 103.62. Therefore it can be inferred that the HPLC method is accurate in all concentration ranges (21).

*Precision*

The inter day and intraday precision results are depicted in the Table 2. respectively. The results indicate that the given method has sufficient precision as indicated by the corresponding values of %R.D.S and %recovery (21).

*Limit of detection and limit of quantification*

The LOD and LOQ for Rivastigmine were found to be 0.038 and 0.12 µg/mL. ng/mL. thus the device can measure the concentrations above 0.12 µg/mL with sufficient precision.

*Microparticles drug content*

The amounts of RHT and the surface drug for the microparticle groups are reported in Table 4. As was expected, higher drug concentrations of the solution injected into the spray dryer, resulted in increase of the drug loading and the surface drug.

*Size and morphological study*

Chitosan microparticles were prepared in three groups and various weight ratios of the drug. The FESEM images of the groups are shown in Figure 2. The size and morphological properties for all groups of microparticles are summarized in Table 5.

As can be seen in the images, the more drug concentration of the solution injected into the spray dryer, the more deformed microparticles and distortion. The distortion pattern was changed as well. This changes can be the result of the increase in the initial solution viscosity, which itself is because of the enhanced drug concentration (22).

Spray drying process conditions play an important role in the preparation of the microparticles, that’s why changing the drug concentration of initial solution did not affect the microparticles size. The FESEM images also confirmed that the microparticles size have not changes significantly.

*In-vitro drug release*

The *in-vitro* drug release profiles of microparticles for the all groups over 10 days are shown in Figure 3. The low error bars of the curves indicate the high repeatability of the tests. The initial release rate and the total release of Rivastigmine from microparticles are expected to be in direct relation with drug loading. For example, the burst release of the first group (7% drug loading) and the second group (29% drug loading), were 50% (over 9 h) and 61% (over 6 h), respectively. The differences between the burst releases of these two groups can be attributed to the higher surface drug of the second group. The Rivastigmine release rate of the second group (0.6714 µg/h) is higher than the first (0.5817 µg/h). The drug release from the first and second groups of microparticles continued till 72 h (83%) and 48 h (90%), respectively. The drug release rate was enhanced by increasing the microparticles drug loading, the reason is that the chemical potential (concentration gradient) is higher for the second group.

An opposite result observed for the third group. In this group the amount of drug loading is (42%), which is more than the other groups. But the amount of burst release (27% in 6 h) and the drug release rate (0.4830 µg/h) have significantly decreased. In this group, after 10 days, the drug release was 74% and still increasing.

This phenomenon is caused by the high and deep distortions on the surface of the microparticles which allows smaller particles to stick in the pores of other particles (as shown in Figure 2.

which reduces the total diffusion surface area. Finally, microparticles of the second group were selected for application in transdermal drug delivery System.

*FTIR analysis*

Since the spray drying process is performed at high temperatures, the FTIR analysis is used to evaluate the potential degradation of the drug and the polymer. Figure 4. Shows the FTIR spectra for chitosan, Rivastigmine, chitosan microparticles and freeze-dried chitosan-drug solution.

Taking into account the chemical structure of the Rivastigmine, which possesses the absorption bonds close to 1726 cm^–1^ and 1399 cm^–1^ are corresponded to ester functional group and C=C bands (23, 24).

Chitosan FTIR spectrum displays a series of very sharp absorption bonds. Briefly, the bending absorption of the N-H bonds of the amine-I and the stretching absorption of C-O-C ether bonds give peaks at 1654 cm^–1^ and 1154 cm^–1^, respectively (23, 24).

According to the FTIR spectrum of chitosan microparticles, chitosan and Rivastigmine functional groups peaks are visible at the same wavelength ranges. On the other hand, the freeze-dried chitosan-drug solution and microparticles spectra are mainly the same, which confirms that spray drying process does not degrade the drug.

*Adhesion properties*

The transdermal patches with microparticle to dry adhesive ratios of 5, 10 and 15%, and the RHT patch containing 5.1% Rivastigmine salt (equivalent to the drug content of the 15% patch) were prepared. The adhesion properties of transdermal patches (MPL and 5.1% RHT patches) are shown in Table 6. According to Table 6. Adhesion properties of the patches decrease with the increase in the microparticles contents. The reason behind this decrease in the adhesion properties can be the reduction in the concentration of adhesive in the interface. Considering the similarity of the patches in backing layer, release liner, adhesive material and TDDS thickness, the only parameter that made the patches to have different adhesion properties, can be the difference between microparticle amount of the MPL and RHT patches (25).

For a TDDS, the surface area is of importance. The best patches are the ones that can reach the therapeutic window with the smallest surface area. Among MPL patches, the 15% patch by having more drug content per unit area of the patch, and still having similar adhesion properties was selected and compared to the 5.1% RHT patch from the permeation point of view.

*In-vitro permeation study*

The cumulative percentage of drug permeated through the cellulose membrane placed in Franz diffusion cell was measured for *in-vitro* permeation studies. The cumulative release percentages of the 5.1% RHT and the 15% MPL patches are shown in Figure 5. The tests were accomplished three times for each sample and lasted seven days. The cumulative released Rivastigmine percentage of the 15% and 5.1% RHT patch over 6 days were around 82% and 54%, respectively. The burst release of the patch was 20% over 6 hours. The accumulative (over 6 days) and burst release (over 6 h) for the 5.1% RHT patch were 50 and 11%, respectively. The cumulative drug release of the 5.1% RHT patch, unlike the 15% MPL patch, did not stop after 6 days.

The over slow release rate of the 5.1% RHT patch might be because of the crystallization of Rivastigmine salt in the acrylic adhesive, which is the result of very low solubility of the salt in the acrylic adhesive (26, 27). In order to diffuse in the acrylic adhesive, the crystallized drug have to dissolve in the adhesive first, and since the dissolution rate of crystallized drug in the acrylic adhesive is much lower than the drug diffusion rate, it releases from the patch very slowly. This over slow drug release rate from the RHT patch, lots of drug in the patch would be wasted after application period. Moreover, a large area of the patch is required to provide therapeutic window. Therefore, using microparticles in the 15% MPL patch makes it more efficient than the RHT patch.

*Morphological evaluation of microparticles in the adhesive*

The FESEM images of dispersed group 2 of microparticles in the acrylic adhesive can be seen in Figure 6. 

The Comparison of Figure 6. and Figure 2. Images shows that the size and the morphology of the microparticles were not affected by TDDS preparation process.

## Conclusion

The present study can be considered as an innovative treatment for Alzheimer’s disease. Although the transdermal drug delivery has its own advantages, the short application period, as a result of low drug solubility in adhesives, is still a major limitation in regards of the patient compliance. In this study, Rivastigmine loaded chitosan microparticles as drug reservoirs, were prepared by spray drying method to inhibit the drug crystallization. Transdermal patch containing the microparticles was prepared and evaluated. In comparison to a microparticles-free patch of Rivastigmine, the prepared TDDS showed an excellent prolong drug release. The 6-day-prepared TDDS can be considered as an alternative for one week application of 6 Exelon patches.
